# Development and Characterization of New Energetic Composites Based on HNTO/AN Co-Crystal and Nitro-Cellulosic Materials

**DOI:** 10.3390/polym15071799

**Published:** 2023-04-06

**Authors:** Hani Boukeciat, Ahmed Fouzi Tarchoun, Djalal Trache, Amir Abdelaziz, Redha Meziani, Thomas M. Klapötke

**Affiliations:** 1 Energetic Materials Laboratory (EMLab), Teaching and Research Unit of Energetic Processes, Ecole Militaire Polytechnique, BP 17, Bordj El-Bahri, Algiers 16046, Algeria; 2 Energetic Propulsion Laboratory, Teaching and Research Unit of Energetic Processes, Ecole Militaire Polytechnique, BP 17, Bordj El-Bahri, Algiers 16046, Algeria; 3 Department of Chemistry, Ludwig Maximilian University, Butenandtstrasse 5–13 (D), D-81377 Munich, Germany

**Keywords:** nanostructured nitrocellulose, cocrystal, energetic composite, thermal behavior, decomposition kinetics

## Abstract

To develop advanced cellulose-based energetic composites, new types of high-energy-density formulations containing hydrazine 3-nitro-1,2,4-triazol-5-one (HNTO)/ammonium nitrate (AN) cocrystals combined with nitrocellulose or nanostructured cellulose nitrate (NC and NMCC) were experimentally characterized. The prepared energetic formulations were analyzed in terms of their physicochemical properties, mechanical sensitivities, structural features, and thermal behavior. Their heats of combustion and theoretical energetic performance were assessed as well. Experimental results exhibited the inherent characteristics of the designed NC@HNTO/AN and NMCC@HNTO/AN, including improved density, specific impulse, and impact sensitivity compared to their raw compounds. Besides that, thermo-kinetic findings revealed that the as-prepared insensitive and high-energy-density composites undergo two exothermic decomposition processes, and that NC@HNTO/AN has higher thermal activity. The present study demonstrated the outstanding characteristics of the new composites and could serve as a reference for developing more advanced cellulose-based energetic formulations.

## 1. Introduction

Nowadays, research in energetic materials focuses on the development of modern energetic composites [1]. Through the progress of new high-energy and insensitive composites with elevated density, several issues have been addressed in recent years [1,2]. However, the most recently elaborated energetic formulations are still unable to substitute those actually employed in defense systems due to various drawbacks, such as incompatibility issues, poor thermal and physical stability, inappropriate energetic performance, and high cost, which restrict their further applications [3,4]. As a typical energetic material, nitrated cellulose (NC) remains the most widely used energetic component in different military and civilian applications owing to its outstanding features, including excellent mechanical strength, flammability, a rapid drying rate, and compatibility with several additives [5]. Nonetheless, the long-term experience with conventional NC has demonstrated some of its shortcomings, encompassing high friability, brittleness at low temperatures, and high shock sensitivity [6,7]. In order to overcome these drawbacks associated with the use of traditional NC, numerous approaches to producing alternative cellulose-based energetic materials have been proposed during the past ten years [8,9]. The first procedure adopted consisted of the chemical functionalization of cellulose with explosophoric functional chemical groups to obtain energetic biopolymers with good performances, such as 1-azido-2-hydroxypropyl cellulose ether, butyl- and methyl-nitrates of cellulose, azidodeoxycellulose, and azidodeoxycellulose nitrate [6,9]. Another interesting methodology is based on the structural modification of nitrated cellulose through the elimination of the amorphous parts and partial depolymerization of cellulose precursor [10]. In this context, nitrated microcrystalline cellulose (NMCC), also known as nanostructured nitrocellulose, has attracted a lot of interest and attention in recent years in both academic and industrial domains as a result of its exceptional characteristics [11]. Compared to the common NC, NMCC showed substantial performance improvements, such as high density (1.673 g/cm^3^ for NC and 1.691 g/cm^3^ for NMCC) and nitrogen content (12.68% for NC and 13.17% for NMCC) [12], making it a very interesting alternative candidate for use in advanced energetic materials (EMs) [13,14].

The need for high performance, stable, and insensitive EMs has recently motivated the scientific community to develop several types of energetic compounds, including energy-rich salts, new nitro-compounds, green oxidizers, energetic ionic liquids, and energetic cocrystals [15,16]. Hydrazine 3-nitro-1,2,4-triazol-5-one and ammonium nitrate (HNTO/AN) cocrystals, recently developed by our research group, allow the combination of the features of both energetic ingredients, by which the drawbacks of AN, such as low density (1.725 g/cm^3^), excessive hygroscopicity, and the ordinary temperature solid–solid transition, have been surmounted. According to the literature reports, this approach could significantly enhance the oxygen balance, mechanical sensitivities, density, detonation/combustion performance, and solubility of the formed cocrystal product [17,18]. For instance, HNTO is considered a potential high-nitrogen heterocyclic molecule with good chemical stability and great energy content, making it highly competitive with its NTO precursor and other currently used explosives. Nevertheless, the main problem of HNTO is related to its negative oxygen balance (−38%) [19]. The recent development of energetic cocrystals by incorporating oxidizers, such as ammonium dinitramide (ADN) and ammonium nitrate (AN), into energetic molecules, has been demonstrated to be an efficient technique for enhancing HNTO and AN characteristics [20,21]. More recently, our research group has deeply investigated the preparation and characterization of this optimal cocrystal by employing a molar ratio of HNTO/AN (1:3). They revealed that the HNTO/AN cocrystal has highly desirable characteristics, including great density (ρ = 1.831 g/cm^3^), elevated nitrogen content (*Nc* = 40%) with good thermal stability, improved impact insensitivity (IS = 24 J), and increased enthalpy of reaction, highlighting its promising foundation for use in explosive and propellant compositions [21,22].

The main purpose of this work was to prepare new energetic composites of the HNTO/AN cocrystal and nitrocellulose and nitrated nanostructured cellulose (NC@HNTO/AN and NMCC@HNTO/AN). The chemical compositions of the as-prepared energetic composites were first optimized using thermochemical EXPLO5 software (Version 6.02.06). After that, the optimal NC@HNTO/AN and NMCC@HNTO/AN formulations were thoroughly investigated through the determination of their molecular structures, morphologies, physicochemical properties, and thermal behavior. In addition, their thermo-kinetic parameters were computed using isoconversional models to examine their safety performance for future applications in composite explosives and solid propellants.

## 2. Experimental Section

### 2.1. Materials

NC (*Nc* = 12.61%) and NMCC (*Nc* = 13.08%), with intrinsic viscosities of 950.4 and 290.6 cm^3^/g, respectively, were successfully fabricated at EMLab according to the procedure mentioned elsewhere [12]. HNTO/AN with a molar ratio 1:3 was prepared already following the method reported in our recent paper [21].

### 2.2. Theoretical Design of the Energetic Composites

**Figure 1:**
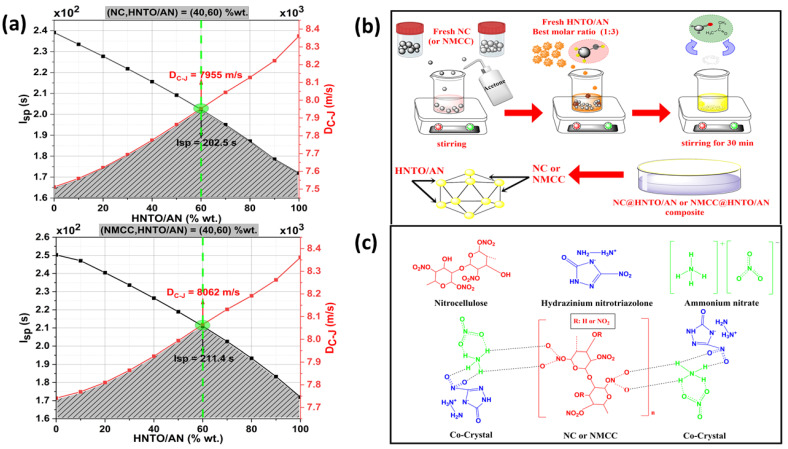
(**a**) Progress of detonation velocity and specific impulse according to the HNTO/AN mass fraction; (**b**) preparation pathway of the composites; (**c**) chemical structures of individual compounds and their composites.

The theoretical calculation of the performances of energetic formulations is essential to optimizing their compositions. Several thermochemical software programs, such as Chemical Equilibrium with Application (CEA-NASA), ICT thermodynamic code, and EXPLO5 (thermochemical program) are available for performing such computations. Before moving further with the development of both NC@HNTO/AN and NMCC@HNTO/AN composites, a theoretical performance investigation using EXPLO5 (version 6.02.06) was required to optimize the chemical compositions and identify two crucial parameters, namely, the specific impulse (*I_SP_*) and detonation velocity (*D_C-J_*). The first criterion, which is the specific impulse, related to the rocket engine efficiency, is computed by supposing isobaric combustion; the detonation parameters were studied with the EXPLO5 code assuming isochoric combustion at the Chapman–Jouguet (CJ) point with the support of the steady-state detonation model using a modified Becker–Kistiakow-–Ki–Wilson equation of state for modeling the system [23]. According to the mass percentage of the HNTO/AN cocrystals, Figure 1a depicts the evolution trend of the predicted *I_SP_* and *D_C-J_*.

### 2.3. Preparation Procedure of the Optimal Composites

Figure 1b illustrates the elaboration pathway of NC@HNTO/AN and NMCC@HNTO/AN composites, for which the amounts of the basic substances were obtained through performance optimization via EXPLO 5, as described above. Each composite contains 40 wt.% of the nitric ester matrix (NC or NMCC) and 60 wt.% of the cocrystal (HNTO/AN). For the preparation, a dried amount of nitrated cellulosic polymer was first dissolved in acetone and stirred for 30 min. Next, the optimized molar ratio of HNTO/AN cocrystal (1:3) was gradually added to the previous mixture under continuous stirring until the solution was completely homogeneous. To remove the solvent, the obtained product was oven-dried under a vacuum at 60 °C overnight.

### 2.4. Characterization Methods

An FEI Quanta 60 scanning electron microscope (SEM), at an accelerating voltage of 5 KV, was used to investigate the morphological features of the elaborated energetic mixtures and their pure compounds. The molecular structure was analyzed using Fourier transform infrared spectroscopy (FTIR, Perkin-Elmer Spectrum 1600) at room temperature. All FTIR spectra were recorded in ATR mode within the wavenumber range of 4000–500 cm^−1^ at a resolution of 4 cm^−1^, for 64 scans. Experimental densities of the studied samples were measured using an electronic densimeter, type AccuPyc 1340 II pycnometer (Micrometrics), at room temperature under helium gas. Density results are presented as the averages of ten measurements. The following formula was used to compare the experimental density ($\rho_{EXP}$) to the theoretical one ($\rho_{TMD}$).

(1) $$∆\rho \left(\%\right)=100\times \frac{/\rho_{EXP}-\rho_{TMD}/}{\rho_{TMD}}$$

where ∆ρ illustrates how the theoretical and experimental composite densities differ.

To assess the thermal decomposition of each developed energetic composite, differential scanning calorimetry (DSC) and thermogravimetry analysis (TGA) were employed. Thermogravimetric analyses, carried out using a Perkin Elmer TG 8000 under a constant nitrogen atmosphere, for about 2–3 mg samples, were performed at a heating rate of 10 °C/min within the temperature range of 50–450 °C. DSC experiments, using a PerkinElmer DSC 8000 analyzer, were conducted under a constant nitrogen gas atmosphere (30 mL/min). For each analysis, 1–2 mg of the dried specimen was used, and the measurements were performed within the temperature interval of 125–250 °C at a heating rate of 10 °C/min. To evaluate the mechanical sensitivities against friction and impact, the standard friction and impact tester instruments were employed. The friction (FS) and impact (IS) sensitivities of the investigated samples were evaluated based on the STANAG 4487 and STANAG 4489, respectively [24,25].

#### Kinetic Calculations

To elucidate the thermal stability of the designed formulations (NC@HNTO/AN and NMCC@HNTO/AN), isoconversional integral models were used, based on non-isothermal DSC results, to predict the thermo-kinetic parameters. As reported by the International Confideration for Thermal Analysis and Calorimetry (ICTAC), for constant conversion (*α*), the temperature is the single variable influencing the reaction rate (Equation (2)).

(2) $$\frac{d\alpha }{dT}=\frac{A_{a}}{\beta }e^{\left(\frac{-E_{a}}{RT}\right)}f\left(\alpha \right)$$

Equation (3) shows the independence between the Arrhenius parameters and the reaction model.

(3) $$g\left(\alpha \right)=\overset{\alpha }{\underset{0}{\int }}\frac{d\alpha }{f\left(\alpha \right)}=\frac{A_{a}}{\beta }\int_{T_{0}}^{T}e^{-E_{a}/RT}dT$$

DSC curves may be exploited to determine the conversion values, which have a maximum value of α = 1.

(4) α=∫t0t(dHdt)dt∫t0tf(dHdt)dt=∆H ∆H total

In these parts, two isoconversional linear methods, specifically, Trache–Abdelaziz-Siwani (TAS) [26] and iterative Kissinger–Akahira–Sunose (it-KAS) [27], as along with the Vyazovkin non-linear isoconversional technique combined with the compensating effect (VYA/CE) [28], were programmed and used to determine the kinetic triplet (*E_a_*, *Log*(*A*), *g*(*α*)).

## 3. Results and Discussion

### 3.1. Determination of the Optimal Compositions of the Energetic Composites

For effective use of the developed energetic formulations in propellant systems and composite explosives, a selection of the most favorable composition of each must be performed. In this research, we optimized two important criteria, the specific impulse (*I_sp_*) and the detonation parameter of Chapman–Juget (*D_C-J_*), using powerful thermochemical software (EXPLO5) to elaborate on the most efficient formulation. Figure 1a exhibits the evolution of the predicted *I_sp_* and *D_C-J_* versus HNTO/AN mass percentage. As can be noticed, an increase in HNTO/AN content, in both formulations, decreases the *I_sp_*, which is due to the negative oxygen balance of HNTO/AN (−19%) leading to incomplete combustion, hence affecting the flame temperature, which is directly proportional to the specific impulse [29,30]. In addition, the intersection of two curves, presented in Figure 1a, presents the optimal composition of an energetic composite. This region exhibits a satisfactory *I_SP_* (202.5 s ≤ *I_SP_* ≤ 211.4 s) and relatively high *D_C-J_* (7955 m/s ≤ *D_C-J_* ≤ 8062 m/s) for the optimized NC (or NMCC)@HNTO/AN (60:40, wt.%) composite. Another interesting finding that can be revealed from Figure 1a is that the NMCC@HNTO/AN composite provides better *I_SP_* and *D_C-J_* than NC@HNTO/AN, highlighting the importance of using NMCC rather than pristine NC to boost the performance. These findings also indicate the relation between the NMCC morphology and its physicochemical characteristics, which could promote the energetic features of the obtained composite. In addition, it is interesting to note that the new elaborated energetic composites have superior *D_C-J_ values* compared to those of triaminotrinitrobenzene (TATB: 7940 m/s), nitroglycerine (NG: 7700 m/s) [31], 1,3-difluoro-2,4,6-trinitrobenzene (DFTNB: 7800 m/s), trinitrotoluene (TNT: 6860 m/s) [32], and other aluminized explosives reported by Keshavarz et al. [33]. In addition, it was found that the optimal NMCC@HNTO/AN composite displayed similar or slightly lower *Isp* than some of the commonly employed double-base propellants (220 s) [34,35], and other NC-based mixtures such as NC/GAP/LLM-105 (207 s) [36] and NC/HMX (232 s) [25]. Overall, these results demonstrate that the newly developed energetic composites can be used as promising systems in futuristic energetic formulations.

### 3.2. Morphological and Chemical Structures

**Figure 2:**
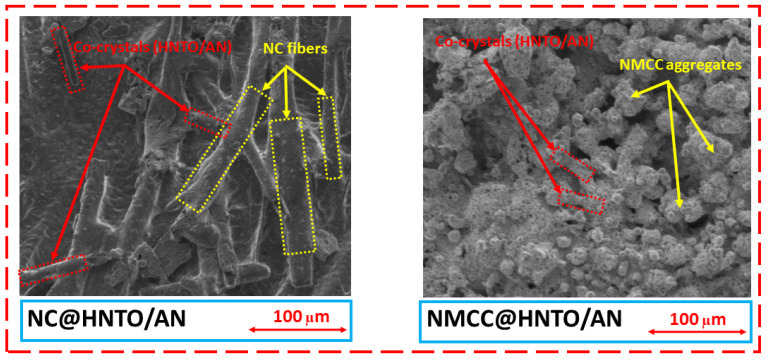
SEM micrographs of the elaborated energetic composites.

**Figure 3:**
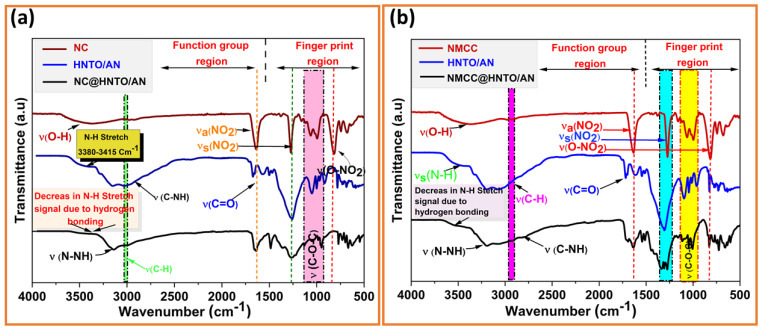
FTIR spectra of (**a**) the NC@HNTO/AN mixture and its pure constituents; (**b**) the NMCC@HNTO/AN mixture and its single constituents.

The morphology characteristics and the chemical structures of the designed NC@HNTO/AN and NMCC@HNTO/AN formulations were examined by SEM and FTIR analyses, respectively, and the obtained findings are illustrated in Figure 2 and Figure 3.

SEM analysis was used to evaluate the morphologies and microstructures of the obtained NC@HNTO/AN and NMCC@HNTO/AN composites, and the acquired micrographs are depicted in Figure 2. It can be perceived that the morphology of NC@HNTO/AN is different from that of NMCC@HNTO/AN. As reported in our previous works, NC has large fibers. NMCC has tiny rods with some visible aggregates, and the HNTO/NA cocrystal particles display a rod-like shape and a smooth surface [21,37]. The structured morphologies of HNTO/AN and NC can be seen in the NC@HNTO/AN composite, indicating the strong interactions between NC chains and the rod-shaped HNTO/AN cocrystals. On the other hand, the NMCC@HNTO/AN formulation displayed cocrystal rods encapsulated in an interconnected system with some aggregates, revealing that the utilization of structurally modified NMCC rather than ordinary NC results in more evenly distributed HNTO/AN cocrystal, which can offer better energetic performance. Such improved dispersion is mainly related to the good interfacial contact between the two components of the energetic formulation, which could enhance the thermolysis process of the composite, as will be shown later in the next parts.

To further elucidate the above results, the experimental density of each of the developed energetic formulations, which is an important factor that may affect their energetic properties, was measured. The density of NMCC@HNTO/AN (1.801 ± 0.003 g/cm^3^) was found to be higher than that of NC@HNTO/AN (1.790 ± 0.003 g/cm^3^), which is even greater than those of pure NC (1.671 ± 0.004 g/cm^3^) and NMCC (1.694 ± 0.004 g/cm^3^). These findings are consistent with SEM results and confirm the benefits of using NMCC to produce outstanding high-density composites. In addition, it is revealed that the experimental densities of the as-prepared energetic composites were comparable to their calculated theoretical values—the ∆ρ was lower than 2%, which indicates the efficiency of the performed mixing process to achieve an adequate homogenization of the resultant energetic composites, hence avoiding the gaseous bubbles’ contamination [30,38]. In addition, it is worth noting that the energetic formulation based on the HNTO/AN cocrystal and NMCC binder has a greater density than that of the widely used double-base rocket propellants [39] and is almost the same as those of some recently reported energetic composites [40].

FTIR experiments were conducted to identify the chemical structures of the designed energetic composites and to examine if there were shifts, appearances, or disappearances of bands between the basic ingredients (NC, NMCC, and HNTO/AN) and the final composites (NC@HNTO/AN and NMCC@HNTO/AN). As can be observed from the spectra plotted in Figure 3, both NC@HNTO/AN and NMCC@HNTO/AN formulations exhibited the typical bands of nitrated cellulose chains at 3500–3470 cm^−1^ and 2900 cm^−1^, which are attributed to the O-H and C-H stretching, respectively, and the absorption bands of NO_2_ and O-NO_2_ in the spectra region of 1800–500 cm^−1^. Moreover, the characteristic groups of the HNTO/AN cocrystal were also detected in the spectra of NC@HNTO/AN and NMCC@HNTO/AN composites, such as the C=O stretching at 1680 cm^−1^, the asymmetric and symmetric C-NO_2_ stretching at 1320 cm^−1^, the primary N-H stretching of the hydrazine group at 3380–3400 cm^−1^, and the N-H of the triazole circle at 2730 cm^−1^ [21,41]. In addition, the decreases in intensity of the OH and NH bands suggest the presence of hydrogen bonds between nitrated cellulosic chains and HNTO/AN cocrystals. For instance, Figure 1c shows an initial attempt to assess the hydrogen bonding interactions between the negatively charged nitro group of HNTO and the positively charged hydrogen atom of AN in the cocrystal, and between the positively charged hydrogen atom of the cocrystal and the negatively charged nitro group of the nitrated cellulosic matrix. On this subject, it is important to mention that deep research is in progress to further elucidate the eventual interactions between the components of the as-prepared energetic formulations using thermogravimetry coupled with infrared spectroscopy (TGA/FTIR) and density functional theory (DFT). The outcomes demonstrate that no modifications in the molecular structures of HNTO/AN, NC, or NMCC were caused by the elaboration procedure, confirming their good compatibility.

### 3.3. Assessment of the Thermal Behavior

**Figure 4:**
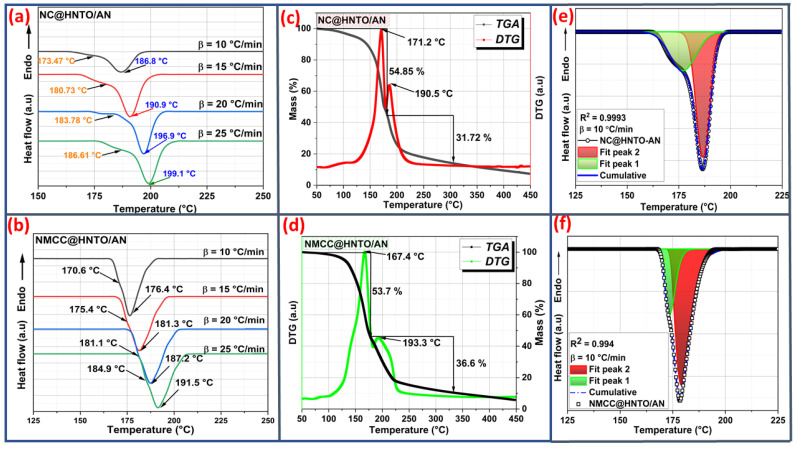
(**a**,**b**) DSC thermograms at various heating rates of the energetic composites; (**c**,**d**) TGA/DTG curves of NC@HNTO/AN and NMCC@HNTO/AN at *β* = 10 °C/min; and (**e**,**f**) deconvolution illustrations of the DSC curves at *β* = 10 °C/min.

Thermal properties are broadly necessary for the examination of energetic materials, which influence their application and safety performance. Therefore, the thermal characteristics of the designed energetic formulations were evaluated by TGA, and the TGA/DTG curves recorded over the temperature range of 50–450 °C were plotted in Figure 4c,d. It was previously discussed in our research papers that nitrated cellulosic polymers exhibit one mass loss stage (≥95%)—at 205.9 °C for NC and 198.7 °C for NMCC, whereas HNTO/AN undergoes a one-step decomposition process at 239.3 °C with 95% weight loss [29,42]. In the case of the developed energetic formulations, it is evident from Figure 4c,d that both NC@HNTO/AN and NMCC@HNTO/AN composites exhibit two decomposition stages. The first step, noted at 165–175 °C with weight loss amounts of 54.85% for NC@HNTO/AN and 53.7% for NMCC@HNTO/AN, is related to the primary homolytic decomposition of nitrated cellulosic materials via splitting of energetic O-NO_2_. However, the second process, which occurred at 180–190 °C with total weight loss levels of 31.72% for NC@HNTO/AN and 36.6% for NMCC@HNTO/AN, is attributed to the decomposition of the HNTO/AN cocrystal, for which more details about the decomposition mechanism of this latter can be found in the work of Abdelaziz et al. [43]. It is worth noting that both energetic formulations lose almost 90% of their initial weight at 450 °C. In addition, the energetic composite containing NMCC as an energetic matrix had a lower mass-loss temperature than that based on NC. According to the study of Sovizi et al. [44], this behavior is due to the quick heat transfer that acts on the thermolysis process when the particle size is reduced. This finding was also stated by Dobrynin et al. [45] and Chen et al. [46], who mentioned that substituting NC with its nano- or microsized derivatives increases the thermal reactivity and combustion performance of the obtained energetic composites. In addition, the thermal decomposition process of the HNTO/AN cocrystal was shifted from 239.3 °C to approximately 190 °C, which was caused by the reactive radicals and energy produced by the primary thermolysis of nitrated cellulosic matrices that accelerate the thermolysis process of the cocrystal.

On the other hand, DSC characterizations were conducted at various *β* to recognize the different endothermic/exothermic decomposition processes and to further investigate the difference between the thermal behavior of the produced formulations. Figure 4a,b shows the acquired DSC curves of the elaborated energetic formulations at various values of *β*, and the onset and maximum decomposition temperatures (*T*_onset_ and *T*_peak_) and the reaction enthalpy (∆*H*) are given in Table 1. Based on Figure 4a,b, both NC@HNTO/AN and NMCC@HNTO/AN composites exhibited two consecutive exothermic events, which are attributed to the nitrate esters’ decomposition stage and the principal thermolysis of the HNTO/AN cocrystal, respectively. In addition, it was found that the obtained decomposition stages are highly dependent on the *β*, and hence are kinetic phenomena due to the fact that a higher heating rate lowers the response time of particles’ heating transfer and increases the decomposition temperatures. In addition, it is evident from the results summarized in Table 1 and Figure 4a,b that the decomposition temperatures of NMCC@HNTO/AN are noticeably lower than those of NC@HNTO/AN. As previously shown by SEM analysis, this behavior is induced by the improved interfacial connection between HNTO/AN particles and the NMCC matrix, which facilitates heat and mass transfers inside the formulation. This result is supported by the fact that the values of ∆*T* given in Table 1—which represent the differences between the *T*_peak_ and the *T*_onset_ of the various thermal decompositions—are notably lower in the case of the NMCC@HNTO/AN formulation compared to those of the NC@HNTO/AN composite, providing evidence for the advantage of replacing the traditional NC with the emergent NMCC. Another intriguing feature that can be deduced from the obtained DSC findings is the slowing of the decomposition peaks of HNTO/AN cocrystal, which suggests that nitrate esters enhance the propagation of the exothermic event [47,48]. Additionally, it was also found that NMCC@HNTO/AN provides greater overall enthalpy of decomposition than the NC@HNTO/AN composite (1697.1 J/g vs. 1415.8 J/g). This result demonstrates the benefits of using nitrated microcrystalline cellulose in improving the energetic features of the obtained cellulose-rich composite. It is also interesting to note that the newly obtained NMCC@HNTO/AN and NC@HNTO/AN formulations exhibited comparable thermal decompositions to some previously studied NC-based composites, such as NC/HMX (*T_peak_* = 168.2 °C, *β* = 10 °C/min) [25], NC/CL-20 (*T_peak_* = 170.9 °C, *β* = 10 °C/min) [49], and NC/2,2,2-trinitroethyl-nitrocarbamate (*T_peak_* = 166.4 °C, *β* = 10 °C/min) [50]. According to the obtained thermal outcomes, it can be concluded that both the HNTO/AN cocrystal and nitrated cellulose-rich polymers may influence the thermal behavior of each other via a synergistic effect.

### 3.4. Determination of the Kinetic Parameters

Due to the prospect of thermal runaway in nitrate esters-based composites, the study of their thermal decomposition kinetics is needed to understand their thermal reactivity and control their thermal stability. Hence, the obtained non-isothermal DSC results at various *β* were exploited to calculate the key kinetic parameters (*E_a_*, *Log*(*A*), and g(α)). It is worth mentioning that the lowest energy required to initiate a reaction is typically represented by *E_a_*; however, the rate at which molecules collide in a given time unit is specified by *Log*(*A*). Broadly, it is possible to determine *Log*(*A*) by a model-free method named the compensating effect [48,51].

**Figure 5:**
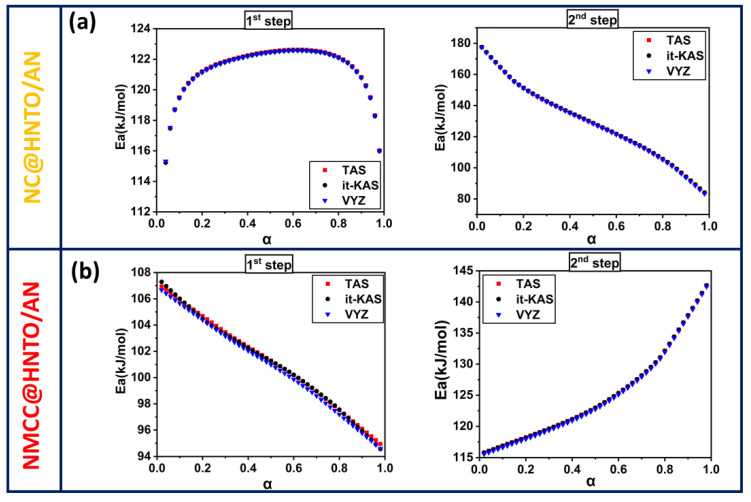
Activation energy progress as a function of conversion for the different stages of decomposition of (**a**) NC@HNTO/AN; (**b**) NMCC@HNTO/AN.

**Figure 6:**
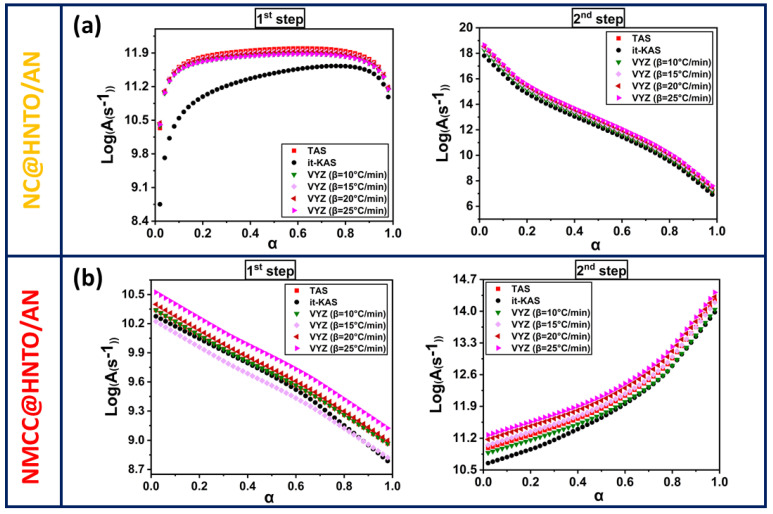
Evolution of the decimal logarithm of the pre-exponential factor as a function of conversion for the different stages of decomposition of (**a**) NC@HNTO/AN; (**b**) NMCC@HNTO/AN.

In order to deconvolute the DSC peaks of the designed NC@HNTO/AN and NMCC@HNTO/AN composites, the asymmetric Frazer–Suzuki function was used, since it is considered as the most popular technique for adjusting multi-step kinetic phenomena [52]. Therefore, Figure 4e,f show examples of the deconvolution of the overlapped DSC peaks, and the experimental data are well matched at all *β* with a high correlation coefficient (*R*^2^ ≥ 0.98). The kinetic parameters for each thermolysis step were then calculated using three isoconversional techniques (TAS, it-KAS, and VYA/CE) based on the deconvoluted DSC data. The dependencies of the Arrhenius factors as a function of conversion on each thermolytic step of the designed NC@HNTO/AN and NMCC@HNTO/AN composites are plotted in Figure 5 and Figure 6, and Table 2 lists the mean values of *E_a_* and *Log*(*A*) along with their corresponding errors, and the most probable integral model g(α). The main finding is that the *Ea* and *Log*(*A*) values calculated by the three used isoconversional approaches for both composites (NC@HNTO/AN and NMCC@HNTO/AN) are in line with each other, pointing out the excellent coherence of the performed calculations. The high R^2^—more than 0.9993—is another indication of the reliability of the calculated kinetic parameters using the linear TAS and it-KAS approaches [28,53].

**Figure 7:**
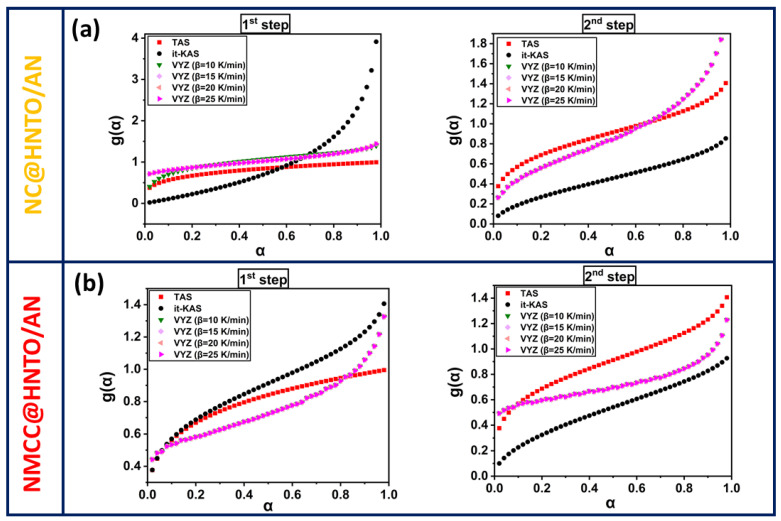
Evolution of the integral reaction model as a function of conversion for the different decomposition stages of (**a**) NC@HNTO/AN; (**b**) NMCC@HNTO/AN.

Figure 5 and Figure 6 illustrate the progress of *Ea* and *Log*(*A*) versus *α* for the thermal decomposition processes of NC@HNTO/AN and NMCC@HNTO/AN formulations. The conversion was fixed within the range of 0.02–0.98 to prevent the intrinsic errors associated with the initial and end periods. The computed results reveal that the applied kinetic methods provide similar values and a consistent trend for each examined composite. Another observation is that, for each decomposition event, the evolution tendencies of *Ea* and *Log*(*A*) with conversion are comparable, which is consistent with the energy compensation effects [51]. According to Figure 6 and Figure 7, the decomposition steps of the elaborated composites display various Arrhenius parameter trends, suggesting that their kinetic trends correspond to the decomposition of the monomolecular NC, NMCC, and HNTO/AN components. Regarding the first step of decomposition, it can be clearly revealed from Figure 6 and Table 2 that the mean values of *Eα* for NC@HNTO/AN and NMCC@HNTO/AN are, respectively, 121 and 101 kJ/mol, which are lower than those of pure NC (155 kJ/mol) and NMCC (140 kJ/mol) matrices [54,55]. The same kinetic behavior was reported by Jain et al. [56] and Li et al. [57]. They found an acceleration in nitrate esters’ thermolysis when AP was added. In this thermolysis process, which is attributed to the thermolytic splitting of energetic O-NO_2_ functions, NC@HNTO/AN exhibits notable increases in *Ea* and *Log*(*A*) values until *α* = 0.4, followed by a stabilization phenomenon, whereas NMCC@HNTO/AN shows the opposite trend. These findings demonstrate that this thermolysis process starts much more quickly in the case of NMCC@HNTO/AN, which is caused by the large amount of thermally unstable O-NO_2_ of NMCC with regard to NC, which promotes heat accumulation and the formation of hot spots within the composite. For the second decomposition process, assigned to the simultaneous decomposition of the HNTO/AN cocrystal, the evolution profiles of *Ea* and *Log*(*A*) versus conversion for the two prepared energetic composites are different. Furthermore, NMCC@HNTO/AN (124 KJ/mol) presented slightly lower average activation energy than that of NC@HNTO/AN (128 KJ/mol), both of which are higher than that of the HNTO/AN cocrystal (121 KJ/mol). These findings demonstrate the effects of nitrate ester cellulosic polymer (NC or NMCC) on reducing the reactivity and the rate of decomposition of the HNTO/AN cocrystal.

On the other hand, the prediction of g(*α*) from the used isoconversional approaches is another crucial factor to consider. Figure 7 shows the evolution of g(*α*) vs. α, and Table 2 presents the mathematical formulas of the models. It is worth mentioning that the Vyazovkin method could not provide the mathematical expression, but the employment of the compensation effect concept allows for obtaining experimental g(*α*). Based on the TAS method, the decomposition of NC@HNTO/AN follows a random nucleation process of Avrami–Erofeev and power-low nucleation in the first and second stages, respectively. However, NMCC@HNTO/AN decomposes following a random nucleation process of Avrami–Erofeev for decomposition steps. The same models were already obtained for the HNTO/AN cocrystal, as mentioned in our previous work [21], revealing that the nitrate esters thermolysis does not affect the reaction model. The obtained thermokinetic parameters provide outstanding information about the significance of preparing new energetic formulations based on nitrated cellulosic matrices and HNTO/AN cocrystals for effective use in high-performance composite explosives and solid propellants.

### 3.5. Sensitivity Features

Mechanical sensitivities of the fabricated energetic mixtures toward impact and friction were evaluated and compared to those of their raw components in order to investigate their safety features, and the obtained results are listed in Table 3. According to the UN Recommendations on the Transport of Dangerous Goods [58], both the designed energetic composites and their basic materials are friction insensitive (FS ≥ 350 N). As a result of the synergistic action and higher interfacial contact between the HNTO/AN cocrystal and NC (or NMCC), the impact sensitivities of NC@HNTO/AN and NMCC@HNTO/AN were revealed to be comparable to that of HNTO/AN cocrystal, but remarkably better than those of NC and NMCC binders. Therefore, it can be concluded that the uniform and homogenous distribution of the HNTO/AN cocrystal within the nitrated cellulosic matrix would decrease the hot spot generation, which would reduce the sensitivity of the obtained energetic formulation. Accordingly, the produced NC@HNTO/AN and NMCC@HNTO/AN mixtures have stable physical properties, making them interesting candidates for military applications.

## 4. Conclusions

In the current work, new energetic formulations based on the HNTO/AN cocrystal and nitro-cellulosic matrices (NC and NMCC) were effectively designed using the casting process. Before that, theoretical performance calculations using the EXPLO5 software were performed to evaluate the optimal compositions of NC@HNTO/AN and NMCC@HNTO/AN mixtures. SEM and FTIR findings and density experiments of the developed composites showed appropriate homogeneity with good dispersion of HNTO/AN cocrystal across the nitro-cellulosic chains. Furthermore, the NMCC@HNTO/AN composite exhibited poorer thermal and kinetic parameters compared to those of NC@HNTO/AN baseline, though a higher reaction enthalpy was obtained, demonstrating the positive effect of using nitrated nanostructured cellulose instead of native nitrocellulose. In addition, the thermolysis of the HNTO/AN cocrystal was also increased by the reactive radicals released from the decomposition of the nitrate esters, which may improve the combustion rate of the formulation. In addition, the computed isoconversional kinetic approaches indicate that the elaborated formulations decompose according to different mechanisms, such as a low-power nucleation model and a random Avrami–Erofeev nucleation process. In light of these results, it can be deduced that the designed NC@HNTO/AN and NMCC@HNTO/AN composites can be seen as outstanding candidates for advanced composite explosives and solid propellants.

## Figures and Tables

**Table 1:** DSC data of the designed energetic formulations determined at *β* = 10 °C/min.

Samples	1st Decomposition				2nd Decomposition				
	*T*_onset_ (°C)	*T*_pic_ (°C)	∆*T* * (°C)	∆*H* (J g^−1^)	*T*_onset_ (°C)	*T*_pic_ (°C)	∆*T* * (°C)	∆*H* (J g^−1^)	∆*H_T_* (J g^−1^)
NMCC@ HNTO-AN	167.2	172.8	5.6	315.66	171.6	176.4	4.8	1381.31	1697.1
NC@ HNTO-AN	160.1	173.5	13.49	379.85	175.1	186.8	11.7	1035.89	1415.8
HNTO/AN	/	/	/	/	235.1	239.3	241.5	1272.7	1272.7 [29]

* ∆*T* = *T*_peak_ − *T*_onset_; ∆*H_T_*, total heat release.

**Table 2:** Kinetic parameters of the prepared energetic composites.

Sample	Isoconversional Method		*Eα* (kJ/mol)	*Log*(*A*(s^−1^))	g(*α*)
NC@HNTO/AN 1st step	TAS		121.10 ± 11.30	11.81 ± 1.75	A_4_ *=* [−*ln*(*1* − *α*)]*^1/4^*
	it-KAS		121.01 ± 11.25	11.22 ± 1.74	P_1/4_ *= α ^1/4^*
	VYA/CE	*β* = 10 °C/min	121.62 ± 11.65	11.72 ± 1.20	/
		*β* = 15 °C/min		11.77. ± 1.20	/
		*β* = 20 °C/min		11.75 ± 1.20	/
		*β* = 25 °C/min		11.71 ± 1.20	/
NC@HNTO/AN 2nd step	TAS		129.20 ± 8.70	12.91 ± 1.56	P_1/4_ *= α ^1/4^*
	it-KAS		128.91 ± 8.60	12.90 ± 1.57	G_7_ *=* [*1* − (*1* − *α*)*^1/2^*]*^1/2^*
	VYA/CE	*β* = 10 °C/min	128.62 ± 8.30	12.88 ± 1.31	/
		*β* = 15 °C/min		12.89 ± 1.32	/
		*β* = 20 °C/min		12.88 ± 1.34	/
		*β* = 25 °C/min		12.88 ± 1.32	/
NMCC@HNTO/AN 1st step	TAS		101.14 ± 8.85	9.67 ± 1.25	A_4_ *=* [−*ln*(*1* − *α*)]*^1/4^*
	it-KAS		101.08 ± 8.90	9.61 ± 1.24	G_8_ *=* [*1* − (*1* − *α*)*^1/2^*]*^1/2^*
	VYA/CE	*β* = 10 °C/min	100.83 ± 8.75	9.67 ± 1.05	/
		*β* = 15 °C/min		9.65 ± 1.04	/
		*β* = 20 °C/min		9.70 ± 1.07	/
		*β* = 25 °C/min		9.64 ± 1.08	/
NMCC@HNTO/AN 2nd step	TAS		125.1 ± 7.95	11.86 ± 1.64	A_4_ *=* [−*ln*(*1* − *α*)]*^1/4^*
	it-KAS		125.05 ± 7.95	12.01 ± 1.65	G_8_ *=* [*1* − (*1* − *α*)*^1/2^*]*^1/2^*
	VYA/CE	*β* = 10 °C/min	124.85 ± 7.73	11.97 ± 0.98	/
		*β* = 15 °C/min		12.01 ± 0.97	/
		*β* = 20 °C/min		12.03 ± 0.96	/
		*β* = 25 °C/min		12.01 ± 0.97	/

**Table 3:** Mechanical sensitivities and densities of the investigated composites.

Sample	$\rho_{EXP}\;(g/{\mathrm{cm}}^{3})$	$\rho_{TMD}\;(g/{\mathrm{cm}}^{3})$	Δρ(%)	IS (J)	FS (N)
HNTO/AN	1.831 ± 0.003	/	/	24	>360
NC	1.671 ± 0.004	/	/	3	350
NMCC	1.694 ± 0.004	/	/	2	350
NC@HNTO/AN	1.790 ± 0.003	1.767	1.3	11	360
NMCC@HNTO/AN	1.801 ± 0.003	1.776	1.4	11	360

## Data Availability

Available data are presented in the manuscript.
